# The Cost-Effectiveness of Digital Health Interventions on the Management of Cardiovascular Diseases: Systematic Review

**DOI:** 10.2196/13166

**Published:** 2019-06-17

**Authors:** Xinchan Jiang, Wai-Kit Ming, Joyce HS You

**Affiliations:** 1 School of Pharmacy, The Chinese University of Hong Kong Shatin China (Hong Kong)

**Keywords:** telemedicine, cardiovascular diseases, stroke, heart failure, myocardial infarction, heart attack, cost-effectiveness, medical economics, decision modeling, systematic review

## Abstract

**Background:**

With the advancement in information technology and mobile internet, digital health interventions (DHIs) are improving the care of cardiovascular diseases (CVDs). The impact of DHIs on cost-effective management of CVDs has been examined using the decision analytic model–based health technology assessment approach.

**Objective:**

The aim of this study was to perform a systematic review of the decision analytic model–based studies evaluating the cost-effectiveness of DHIs on the management of CVDs.

**Methods:**

A literature review was conducted in Medline, Embase, Cumulative Index to Nursing and Allied Health Literature Complete, PsycINFO, Scopus, Web of Science, Center for Review and Dissemination, and Institute for IEEE Xplore between 2001 and 2018. Studies were included if the following criteria were met: (1) English articles, (2) DHIs that promoted or delivered clinical interventions and had an impact on patients’ cardiovascular conditions, (3) studies that were modeling works with health economic outcomes of DHIs for CVDs, (4) studies that had a comparative group for assessment, and (5) full economic evaluations including a cost-effectiveness analysis, cost-utility analysis, cost-benefit analysis, and cost-consequence analysis. The primary outcome collected was the cost-effectiveness of the DHIs, presented by incremental cost per additional quality-adjusted life year (QALY). The quality of each included study was evaluated using the Consolidated Health Economic Evaluation Reporting Standards.

**Results:**

A total of 14 studies met the defined criteria and were included in the review. Among the included studies, heart failure (7/14, 50%) and stroke (4/14, 29%) were two of the most frequent CVDs that were managed by DHIs. A total of 9 (64%) studies were published between 2015 and 2018 and 5 (36%) published between 2011 and 2014. The time horizon was ≤1 year in 3 studies (21%), >1 year in 10 studies (71%), and 1 study (7%) did not declare the time frame. The types of devices or technologies used to deliver the health interventions were short message service (1/14, 7%), telephone support (1/14, 7%), mobile app (1/14, 7%), video conferencing system (5/14, 36%), digital transmission of physiologic data (telemonitoring; 5/14, 36%), and wearable medical device (1/14, 7%). The DHIs gained higher QALYs with cost saving in 43% (6/14) of studies and gained QALYs at a higher cost at acceptable incremental cost-effectiveness ratio (ICER) in 57% (8/14) of studies. The studies were classified as excellent (0/14, 0%), good (9/14, 64%), moderate (4/14, 29%), and low (1/14, 7%) quality.

**Conclusions:**

This study is the first systematic review of decision analytic model–based cost-effectiveness analyses of DHIs in the management of CVDs. Most of the identified studies were published recently, and the majority of the studies were good quality cost-effectiveness analyses with an adequate duration of time frame. All the included studies found the DHIs to be cost-effective.

## Introduction

### Digital Health Interventions

The application of information technology and mobile internet in the health care industry takes the practice of patient care to the era of digital health. Digital health is the convergence of science and technology with health, health care, living, and society [1]. According to the US Food and Drug Administration (FDA), the broad scope of digital health includes a wide range of subsectors: mobile health, telemedicine, telehealth, wearable devices, and personalized medicine [2]. Stakeholders, such as health care practitioners and researchers, have adopted digital health interventions (DHIs) aiming to promote access, reduce costs, personalize medicine, and improve outcomes of patient care. Various types of digital devices or technologies are used to deliver the health interventions, such as the short message service (SMS); mobile app; telephone; video conferencing system; digital, broadband, satellite, wireless, or Bluetooth for monitoring and transmission of physiologic data (telemonitoring [TM]); and wearable medical device [3-6].

### Use of Digital Health Interventions in Cardiovascular Diseases

Cardiovascular diseases (CVDs) cause 17.9 million deaths per year, accounting for 31% of all mortality globally [7]. It is estimated that the global costs of CVDs will rise from US $863 billion in 2010 to US $1044 in 2030 [8]. The potential benefits of DHIs in CVDs were examined in clinical trials. TM was applied in an intensive follow-up of heart failure (HF) patients after discharge in the *TElemonitoring in the MAnagement of Heart Failure* study [9]. Compared with the usual care group, all-cause mortality was significantly lower in the TM group. The number of follow-up days lost to HF-related events was also significantly reduced in the TM group. A randomized controlled trial investigated the effect of CardioFit, an internet-based expert system, in patients with coronary heart disease (CHD) [10]. Patients in the CardioFit group received 5 Web-based tutorials over a period of 6 months for activity planning and tracking and were in contact with an exercise specialist. Physical activity, measured by a pedometer and self-report, was improved over a period of 12 months. A meta-analysis on 51 studies of DHIs in patients with CVDs or risk factors of CVDs reported that DHIs were associated with reduction of cardiovascular event rates and had a positive impact on risk factors for CVDs [11].

### Decision Analytic Model–based Health Technology Assessment

In addition to the improved clinical outcomes of DHIs for CVD patients, evaluating the health economic outcomes is also crucial for clinicians, patients, and third-party payers in deciding the role of DHIs for CVD management. Decision analytic modeling is an approach that synthesizes cost-effectiveness evidence of health technologies and interventions in health technology assessment (HTA) [12]. This approach provides a framework to incorporate relevant clinical probabilities and cost items, simulates outcomes of disease management, and allows cost-effectiveness evaluation of medical interventions. Decision tree and Markov models are 2 commonly used forms of decision analytical modeling in health economic evaluation. In a decision tree, distinct branches are used to represent a potential set of outcomes for the patient cohort managed by an alternative treatment. Outcomes and costs for each branch are combined using branch possibilities to simulate the expected outcomes and costs for the treatment option. In a Markov model, hypothetical patients proceed through different health states over time based on transition probabilities between health states. Outcomes and costs expected by the patient cohort in an alternative treatment group are estimated from subject-time spent in various health states. The confidence level in the output of an economic modeling analysis, in relation to uncertainty in the model inputs, is typically quantified by techniques such as one-way and probabilistic sensitivity analyses. By applying model-based HTA, the cost-effectiveness impacts of various types of health technologies and interventions are compared using findings from corresponding clinical trials [12,13]. The implementation cost of DHIs is usually substantial, and HTA is, therefore, essential to inform the decision makers on the potential impact of the DHIs on both clinical and health economic outcomes [14]. The cost-effectiveness of DHIs is subject to the balance of 3 elements: clinical and economic benefits of DHI, cost of DHI, and payer’s willingness-to-pay (WTP) threshold. A previous review of HTA studies on DHIs indicated that there were few health economic studies of DHIs [15].

### Objective

With increasing publications on the cost-effectiveness of DHIs in CVDs, the purpose of this study is to conduct a systematic review of decision analytic model–based health economic analyses of DHIs for CVD management.

## Methods

### Search Strategy

The investigators developed the search strategies from September to October 2018 to include a wide range of DHIs. Analogously, search terms included different types of CVDs, such as HF, myocardial infarction, and stroke. The literature search was conducted in the following databases: Medline, Embase, Cumulative Index to Nursing and Allied Health Literature Complete, PsycINFO, Scopus, Web of Science, Center for Review and Dissemination, and Institute for IEEE Xplore. A preliminary search found an evident surge of publications on digital technologies in the field of health and medical research starting in the 2000s, and all databases were, therefore, searched back to 2001. A manual search of reference lists of both included studies and relevant systematic reviews was also conducted. Multimedia Appendix 1 provides detailed information about the search terms. This study was registered on PROSPERO with the registration number of CRD42018111473.

### Inclusion and Exclusion Criteria

Full-text journal articles written in English were included if (1) the target population was patients with CVDs, (2) DHIs were aimed to promote or deliver clinical interventions and had an impact on cardiovascular conditions, (3) decision analytic models (including decision tree and/or the Markov model) were applied to evaluate health economic outcomes of DHIs, (4) the interventions were compared with conventional care or other DHIs, and (5) a full-scale health economic evaluation was performed as a cost-effectiveness analysis, cost-utility analysis, cost-benefit analysis, or cost-consequence analysis.

The exclusion criteria included the following: (1) DHIs were only used for recording patients’ information, (2) studies were conducted alongside a clinical trial, (3) quality-adjusted life years (QALYs) were not reported, or (4) the articles were reviews, protocol papers, letters, editorials, conference abstracts, poster presentations with insufficient details, or case reports.

### Study Selection

After removing the duplicates, titles and abstracts were screened for eligibility. The full text of eligible articles was then reviewed for verification of eligibility. The primary search was conducted by one of the investigators (XJ). The abstracts were reviewed by two of the investigators (XJ and WM), independently. Any disagreements were discussed with the third investigator (JY) to reach a consensus. At the final stage of the full-text review, the included articles that met all the predefined criteria were read by all the investigators (XJ, WM, and JY) to confirm inclusion of the articles.

### Data Extraction

A pilot data extraction was conducted by two of the investigators (XJ and WM), independently. Any discrepancy pertinent to data extraction was discussed to reach a consensus. After that, an abstraction form was adopted for guiding further data extraction. The collected information included the following items: (1) general information (including authors, title, country, and publication date), (2) study characteristics (including types of diseases and interventions), (3) methodology (including modeling method, time horizon, and perspective), and (4) summary of quantitative findings and conclusions. The primary outcomes collected were the cost-effectiveness of the DHIs, presented by incremental cost per QALY as the incremental cost-effectiveness ratio (ICER). If the ICER was not available, incremental cost and incremental QALY were assessed.

### Assessment of Methodological Quality

The Consolidated Health Economic Evaluation Reporting Standards (CHEERS) checklist, developed by the International Society for Pharmacoeconomics and Outcomes Research for good reporting of health economic outcomes, was used to assess the methodological quality of each study [16]. The CHEERS checklist included 24 items, and the recommendations were subdivided into 6 categories: (1) title and abstract, (2) introduction, (3) methods, (4) results, (5) discussion, and (6) other. One point was assigned to each item when the quality criteria were fulfilled (and zero points for not entirely conforming to the criteria) to generate a total score (maximum score is 24). The included studies were classified into 4 quality categories: excellent (scored in 100% of the items), good quality (scored between >75% and <100% of the items), moderate quality (scored between >50% and ≤75% of the items), and low quality (scored ≤50% of the items) [17].

Two of the investigators (XJ and WM) independently assessed the quality of each study and assigned the scores based on the CHEERS checklist. Any disagreement was resolved by discussion and consensus with the third investigator (JY).

### Data Analysis and Presentation

The number of studies included and excluded during the selection process was presented in a flowchart. The included studies were categorized by the type of devices or technologies used for DHI delivery. The descriptive characteristics and the study quality of the included studies and ICERs of DHIs were summarized. The DHI was categorized as cost-effective if (1) it was more effective and less costly than the comparator (DHI dominated the comparator) or (2) it was more effective at a higher cost and the ICER was less than the WTP threshold. The cost-effective DHI identified in each included study was presented.

## Results

### Search Results

**Figure 1:**
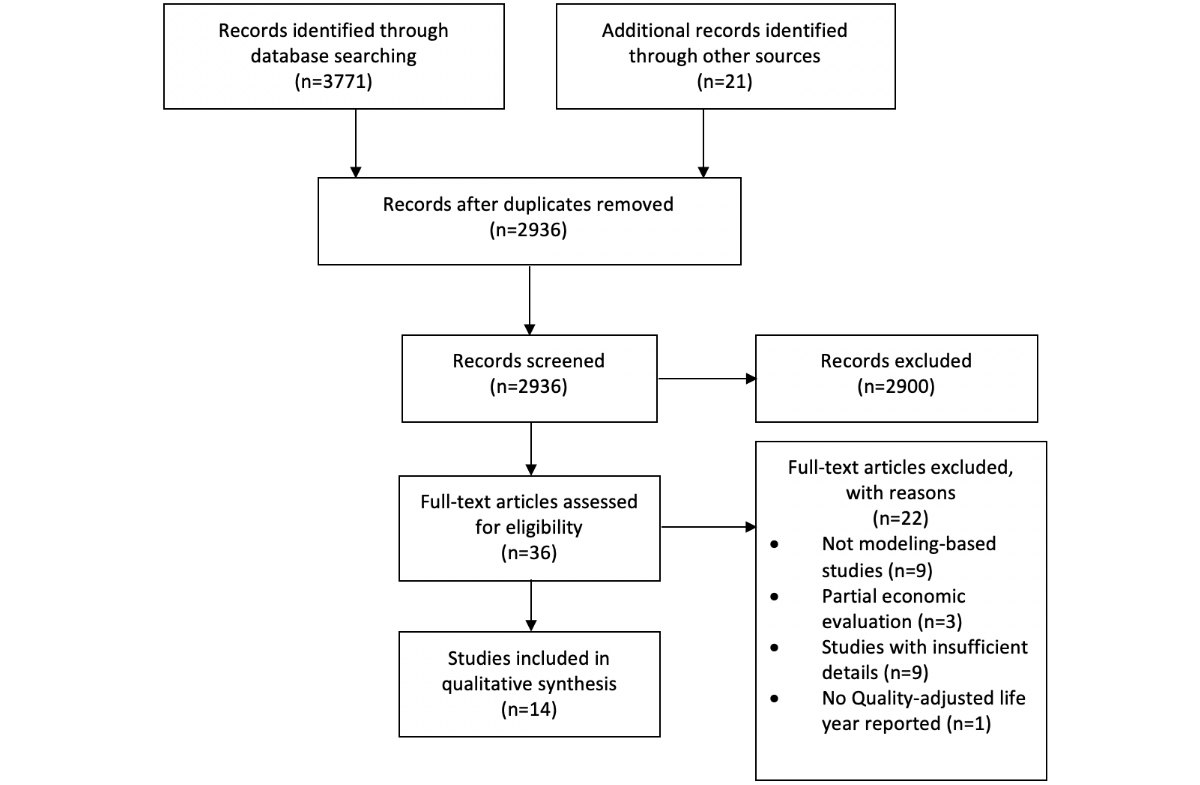
The article selection process according to the Preferred Reporting Items for Systematic Reviews and Meta-Analyses guideline.

The data extraction and selected results are shown in Figure 1. The search retrieved 3771 studies from targeted databases and 21 studies from manual searches. After removal of the duplicates, 2936 articles remained. A total of 14 out of the 36 full-text articles screened according to the Preferred Reporting Items for Systematic Reviews and Meta-Analyses guidelines were included in the review [18].

### Study Characteristics

Table 1 summarizes the characteristics and main health economic outcomes of the included studies.

A total of 10 out of the 14 studies (10/14, 71%) were conducted using the Markov model [19-21,25,27-32] and 4 studies (4/14, 29%) used the decision tree model [22-24,26]. All the 14 studies conducted (14/14, 100%) a cost-utility analysis with cost and QALYs as the outcome measures and 7 studies (7/14, 50%) also performed a cost-effectiveness analysis to include survival as the effectiveness measure [19,20,28-32]. Types of targeted CVDs were CHD [19], HF [20,21,27-31], congenital heart disease (CoHD) [22], stroke [23-26], and sudden cardiac arrest (SCA) [32]. A total of 9 studies (9/14, 64%) were published between 2015 and 2018 [19,20,23,26,28-32] and 5 studies (5/14, 36%) between 2011 and 2014 [21-22,24,25,27]. The types of devices or technologies used to deliver the health interventions were SMS (1/14, 7%) [19], telephone support (1/14, 7%) [20], mobile app (1/14, 7%) [21], video conferencing system (5/14, 36%) [22-26], digital transmission of physiologic data (TM; 5/14, 36%) [27-31], and wearable medical device (1/14, 7%) [32]. The model time horizon in 3 studies (3/14, 21%) was ≤1 year [23,24,26], whereas 10 studies (10/14, 71%) used a time horizon longer than 1 year [19,20,22,25,27-32] and 1 study (1/14, 7%) did not declare the time horizon. In total, 3 studies (3/14, 21%) received funding from the industry [19,25,30], 6 (6/14, 43%) received grants from public organizations [21,23,24,26,27,29], 1 (1/14, 7%) received no funding [22], and 4 (4/14, 29%) did not declare their funding source [20,28,31,32]. All the included studies (14/14, 100%) found the DHIs to be cost-effective: DHIs gained higher QALYs with cost-saving in 6 (6/14, 43%) studies [19,20,22,23,25,32]; DHIs gained QALYs with a higher cost at an acceptable ICER in 8 studies (8/14, 57%) [21,24,26-31]. All the 14 studies conducted a sensitivity analysis, including a probabilistic sensitivity analysis in 10 studies (10/14, 71%) [19,20,22-24,26-29,31] and a one-way sensitivity analysis in 10 studies (10/14, 71%) [22-26,28-32]. A scenario analysis, a subtype of a sensitivity analysis, was performed for the best-case, worse-case or base-case scenarios in 6 (6/14, 43%) studies to inform the optimal scenario for cost-effective use of the DHIs [19,20,26-28,31].

**Table 1:** General characteristics and quality assessment of the included studies.

Technologies or devices for digital health intervention delivery with references and year		Country	Targeted disease	Model type	Perspective	Time horizon	Intervention versus comparator	Incremental cost-effectiveness ratio	Cost-effective strategy (willingness-to-pay where available)	Source of funding	CHEERS^a^ (%) (quality classification)
**Short message service**											
	Burn et al, 2017 [19]	Australia	Coronary heart disease	Markov model	Australia health care system	Lifetime	TEXT ME^b^ program versus UC^c^	TEXT ME program dominated UC	TEXT ME Program (Aus $64,000)	Industry	92 (Good)
**Telephone support**											
	Grustam et al, 2018 [20]	United Kingdom	HF^d^	Markov model	UK third-party payer	0 years	TM^e^ versus UC	€12,479 /QALY^f^	NTS^g^ (€9000)	Not declared	92 (Good)
	Grustam et al, 2018 [20]	United Kingdom	HF	Markov model	UK third-party payer	0 years	NTS versus UC	€8795 /QALY	—^h^	—	—
	Grustam et al, 2018 [20]	United Kingdom	HF	Markov model	UK third-party payer	0 years	NTS versus TM	NTS dominated TM	—	—	—
**Mobile apps**											
	Martín et al, 2014 [21]	Spain	HF	Markov model	Spain health care system	Not declared	CardioManager versus UC	€9.303/ QALY	CardioManager	Public organization	50 (Low)
**Video conferencing system**											
	Mistry et al, 2013 [22]	United Kingdom	Congenital heart disease	Decision tree	UK health service	Lifetime	Telemedicine screening versus direct assessment	Telemedicine screening dominated direct assessment	Telemedicine screening (€20,000)	No funding	71 (Moderate)
	Whetten et al, 2018 [23]	United States	Stroke	Decision tree	US health care payer	90 days	ACCESS^i^ program versus no program	ACCESS program dominated no program	ACCESS program	Public organization	75 (Moderate)
	Nelson et al, 2011 [24]	United States	Stroke	Decision tree	Society	90 days	Telestroke versus UC	US $108,363 /QALY	—	Public organization	92 (Good)
		United States	Stroke	Decision tree	Society	Lifetime	Telestroke versus UC	US $2449 /QALY	Telestroke (US $100,000)	—	—
	Demaerschalk et al, 2013 [25]	United States	Stroke	Markov model	Society	Lifetime	Telestroke versus UC	Telestroke dominated UC	Telestroke (US $50,000)	Industry	79 (Good)
	Nelson et al, 2016 [26]	United States	Stroke	Decision tree	A spoke hospital	Inpatient stay	Telestroke versus UC	US $25,991 /QALY	Telestroke (US $50,000)	Public organization	79 (Good)
	Nelson et al, 2016 [26]	United States	Stroke	Decision tree	A hub hospital	Inpatient stay	Telestroke versus UC	US $47,033 /QALY	—	—	—
**Telemonitoring**											
	Thokala et al, 2013 [27]	United Kingdom	HF	Markov model	National Health Service in England and Wales	30 years	STS HM^j^ versus UC	UC dominated STS HM	TM (€20,000)	Public organization	88 (Good)
	Thokala et al, 2013 [27]	United Kingdom	HF	Markov model	England and Wales	30 years	TM versus UC	£11,873 /QALY	—	—	—
	Thokala et al, 2013 [27]	United Kingdom	HF	Markov model	England and Wales	30 years	Structured telephone support with a human-to-human contact. versus TM	£228,035 /QALY	—	—	—
	Cowie et al, 2017 [28]	United Kingdom	HF	Markov model	UK health care payer	10 years	CardioMEMS vs UC	£19,274 /QALY	CardioMEMS (US $20,000)	Not declared	79 (Good)
	Sandhu et al, 2015 [29]	United States	HF	Markov model	Society	Life time	CardioMEMS versus UC	US $71,462 /QALY	CardioMEMS (US $150,000)	Public organization	88 (Good)
	Schmier et al, 2017 [30]	United States	HF	Markov model	US health care payer	5 years	CardioMEMS versus UC	US $44,832 /QALY	CardioMEMS (US $100,000)	Industry	71 (Moderate)
	Martinson et al, 2017 [31]	United States	HF	Markov model	US health care payer	5 years	CardioMEMS versus UC	US $12,262 /QALY	CardioMEMS (US $50,000)	Not declared	83 (Good)
**Wearable medical device**											
	Healy et al, 2015 [32]	United States	SCA^k^	Markov model	Society	5 years	WCD^l^ versus discharge home	US $26,436 /QALY	WCD (US $50,000)	Not declared	71 (Moderate)
	Healy et al, 2015 [32]	United States	SCA	Markov model	Society	5 years	WCD versus SNF^m^	WCD dominated SNF	—	—	—
	Healy et al, 2015 [32]	United States	SCA	Markov model	Society	5 years	WCD versus in-hospital stay	WCD dominated in-hospital stay	—	—	—

^a^CHEERS: Consolidated Health Economic Evaluation Reporting Standards is a 24-item checklist with a maximum score of 24. Studies that fulfilled 100% of the items were classified as excellent quality, those that fulfilled between >75% and <100% of the items were classified as good quality, those that fulfilled between >50% and ≤75% were classified as moderate quality, and those that fulfilled ≤50% were classified as low quality.

^b^TEXT ME: Tobacco, Exercise, and Diet Messages.

^c^UC: usual care.

^d^HF: heart failure.

^e^TM: telemonitoring.

^f^QALY: quality-adjusted life year.

^g^NTS: nurse telephone support.

^h^Not applicable.

^i^ACCESS: Access to Critical Cerebral Emergency Support Services.

^j^STS HM: structured telephone support with a human-to-machine interface.

^k^SCA: sudden cardiac arrest.

^l^WCD: wearable cardioverter-defibrillator.

^m^SNF: skilled nursing facility.

### Study Quality

The percentage of items fulfilled by each study per the CHEERS checklist is shown in Table 1. The number of studies classified as excellent quality, good quality, moderate quality, and low quality were 0 (0/14, 0%), 9 (9/14, 64%), 4 (4/14, 29%), and 1 (1/14, 7%), respectively. The CHEERS checklist items and detailed quality assessment for each study are listed in Multimedia Appendix 2. In total, 3 CHEERS checklist items were fulfilled by <7 (50%) studies: (1) the abstract was fulfilled in 3 (3/14, 21%) studies [23,24,31], (2) target population and subgroups were fulfilled in 4 (4/14, 29%) studies [19,20,22,29], and (3) characterizing heterogeneity in results was fulfilled in 3 (3/14, 21%) studies [20,26,29]. In total, 7 CHEERS checklist items were fulfilled by all studies (100%): (1) title, (2) background and objectives, (3) comparators, (4) choice of health outcomes, (5) choice of model, (6) analytic methods, and (7) study findings, limitations, generalizability, and current knowledge. The remaining 14 CHEERS items were fulfilled in ≥50% of the studies: (1) setting and location [19-24,27,28,31], (2) study perspective [19,20,22,24,26-29,31,32], (3) time horizon [19,20,22-32], (4) discount rate [19,20,22,24-32], (5) measurement of effectiveness [19,20,24,25,27,31,32], (6) measurement and valuation of preference-based outcomes [19,20,23-31], (7) estimating resources and costs [19-21,23-32], (8) currency, price date, and conversion [19-21,23-32], (9) assumptions [19,20,22,24-29,31,32], (10) study parameters [19,20,22-30], (11) incremental costs and outcomes [19,20,22-32], (12) characterizing uncertainty [19,20,22-25,27-32], (13) source of funding [19,21-27,29,30], and (14) conflicts of interest [19-21,23-32].

### Type of Devices or Technologies for Digital Health Intervention Delivery

#### Short Message Service

The Tobacco, Exercise and Diet Messages (TEXT ME) intervention sent text messages via SMS to CHD patients in addition to their usual physician counseling. The text messages included 4 types of information: general information on heart diseases, nutrition, physical activity, and smoking cessation. A total of 4 text messages were sent per week for 24 weeks. The TEXT ME program was reported to gain 1143 QALYs and save a direct medical cost of Aus $10.56 million over a lifetime horizon for a hypothetical cohort of 50,000 CHD patients in Australia [19].

#### Telephone Support

Structured telephone support is the use of phone calls by specialists, such as nurses, to deliver self-care support and/or management. Nurse telephone support (NTS) for HF patients, managed by a specialist nurse, included monthly assessments of symptoms, current medication, and delivery of timely feedback to physicians and patients. A cost-effectiveness analysis examined the NTS strategy for HF patients in the United Kingdom [20]. NTS gained .14 QALYs and saved €3190 (including direct medical costs) in a 20-year time horizon.

#### Mobile App

CardioManager, a mobile app, was divided into 3 sections to allow the patients to self-manage their heart disease conditions. An informative section that provided medical information and a patient guide. A section that recorded the user’s activities (physical activities and food intake) and health measurements (vital signs). A registry of medications was also included for patients to set alarms for medication administration time. The ICER of the CardioManager was €9.303/QALY (including total direct medical costs) in Spain, yet the study did not specify the time horizon and WTP threshold [21].

#### Video Conferencing System

A total of 5 of the included studies evaluated the cost-effectiveness of delivering specialist consultation services via a video conferencing system for remote patients with heart disease–associated conditions [22-26].

An app of the video conferencing system, using the telemedicine equipment installed in the district hospital, allowed the remote specialist to view live or prerecorded ultrasound images of a pregnant woman and to help the local specialist to identify fetal CoHD. The telemedicine service was reported to gain .042 QALYs and save £30 (total direct medical cost) per child’s lifetime in the United Kingdom [22].

The video conferencing system is applied in the Telestroke network for delivery of neurology care to remote stroke patients. Telestroke operates on a hub-and-spoke system. The spoke facilities are regional hospitals connecting to a hub hospital. The hub hospital serves as the complex stroke care provider and accepts patients from the spoke hospitals. The 4 cost-effectiveness studies of telestroke for management of acute ischemic stroke were all conducted in the United States [23-26]. The Access to Critical Cerebral Emergency Support Services with 12 partner hospitals was reported to save a total direct medical cost of US $4241 and gain .202 QALYs per patient over a period of 90 days [23]. A telestroke system with 1 hub and 8 spokes was found to be accepted as cost-effective with an ICER of US $2449/QALY [24], whereas a network with 1 hub and 7 spokes gained .022 QALYs and saved US $1436 per patient in the lifetime horizon (including both direct medical and indirect costs) [25]. A short-term analysis (duration of inpatient stay) on a network with 2 hubs and 17 spoke facilities reported the network to be accepted as cost-effective (including direct medical costs) for both the hub hospital (ICER=US$47,033/QALY) and the spoke hospital (ICER=US $25,991/QALY) [26].

#### Telemonitoring

TM of HF deterioration indicators (such as changes in blood pressure, intrathoracic impedance, heart rates during rest, and exertion), transmitted to health care providers for review, facilitates early detection of significant changes and allows early intervention for patients with signs of deterioration to prevent emergency admissions and avoid complications. The cost-effectiveness of TM of HF patients postdischarge was examined in the United Kingdom [27]. It was accepted to be cost-effective with an ICER of £11,873/QALY (including direct medical costs) in a 30-year time frame.

CardioMEMS, a wireless pulmonary artery pressure sensor, was the first implantable HF monitoring device approved by the FDA in 2014. The wireless sensor is permanently implanted into the distal pulmonary artery and transmits hemodynamic data to a secure website (as the patient database). Changes in pulmonary artery pressure are used, in conjunction with HF signs, to guide physicians in treatment initiation and adjustment. The cost-effectiveness of CardioMEMS was evaluated in 4 studies (3 studies in the United States and 1 study in the United Kingdom) [28-31]. CardioMEMS was accepted to be cost-effective in the 3 US studies in a lifetime horizon (ICER=US $71,462/QALY including direct and indirect medical costs) [29] and in a 5-year time frame (ICER less than US $50,000/QALY including direct medical cost) [30,31]. Similarly, a 10-year UK analysis also accepted CardioMEMS to be cost-effective with an ICER of £19,274/QALY including direct medical costs [28].

#### Wearable Medical Device

Wearable cardioverter-defibrillator (WCD) is an external device used for monitoring heart rhythm continuously. The WCD alarms the patient by vibration when ventricular arrhythmia (ventricular tachycardia or ventricular fibrillation) is detected. If the patient does not respond to the alarm, the WCD then delivers electric shocks to resuscitate the patient from presumed SCA. The cost-effectiveness of WCD against sudden cardiac death in patients who require temporary removal of the implantable cardioverter-defibrillator was examined [32]. WCD gained higher QALYs and saved total cost (including direct and indirect costs) when compared with discharge-to-skilled nursing facility (by .076 QALYs and US $6681) and in-hospital stay (by .039 QALYs and US $26,001).

## Discussion

### Principal Findings

This is the first systematic review of a decision analytic model–based cost-effectiveness analysis of DHIs in the management of CVDs. This review identified a small but growing body of evidence (14 studies) evaluating the cost-effectiveness of DHIs. These findings were similar to those of a previous review (on cost-effectiveness of telehealth interventions for HF patients) where 7 studies assessed both costs and effectiveness outcomes comprehensively [33].

The assessment of the study quality found that the majority of the methodology items (including comparators, time horizon, choice of models and health outcome measures, and analytical methods; Multimedia Appendix 2) met the requirements of the CHEERS checklist, indicating the scientific rigor of the modeling approaches applied in the included studies. All the 14 included studies were conducted in developed countries, suggesting that DHIs were more ready to be implemented in developed regions. Most of the studies were funded by a public organization (6/14, 43%), followed by the industry (3/14, 21%), showing that both the public sectors and the technology industry have a strong interest to implement cost-effective DHIs for CVD management in the health care system.

The DHIs that showed to be cost saving were SMS for CHD patients [19], telephone support for HF patients [20], wearable medical device for patients at risk of SCA [32], and video conferencing systems for prenatal screening [22] and stroke patients [23,25], whereas the DHIs found to incur higher costs were mobile app for HF patients [21], video conferencing systems for stroke management [24,26], and TM for HF patients [27-31]. The type of DHIs is, therefore, one of the influential cost drivers in the management of CVD patients.

The impact of a technology on health economics is highly subjected to the difference between the technology cost and the change (reduction or increase) in health care resource utilization as a result of the clinical effect of this technology. For instance, the effective technologies improving the survival rate of stroke patients inevitably increased the total treatment costs associated with long-term care for stroke survivors. The cost-effectiveness of the technology was influenced by both the difference in cost and in QALY gained. In this review, all studies (except one in which the time frame was not declared [21]) of DHIs for HF, CHD, CoHD, and SCA patients had used a long-term time frame (ranged from 5 years to lifelong). Both short- and long-term models were used for stroke. As shown by the studies of the Telestroke network, the cost-effectiveness of DHI improved in models with lifelong versus short-term (90 days and inpatient stay) time horizon [24-26]. The time horizon of the model needs to be adequate to capture the QALYs gained by the technology.

### Limitations

This systematic review was limited by the search approach. Only studies written in the English language were included, and limited databases with fixed number keywords were used. Some relevant studies, therefore, might not be identified by this search approach, limiting both the total number and origins of studies (all in developed countries) included. The search was also limited to studies conducted by decision analytic modeling. Despite the fact that the decision analytic model–based methodology allows a cost-effectiveness analysis of multiple treatment strategies (supported by the evidence of corresponding clinical trials) for disease management, economic evaluations conducted alongside clinical trials produce valid and rigorous cost-effectiveness evidence. Further review of cost-effectiveness studies of DHIs for CVDs conducted alongside clinical trials is highly warranted.

### Implications

Despite the small number of model-based health economic analyses on DHIs for CVD management, the majority were ranked to be good quality health economic evaluations and were conducted in the past 5 years. It showed an up-and-rising demand for cost-effective application of DHIs in the management of CVD patients. The development of DHIs has blossomed with the advancement of technology on the internet and mobile devices over the past 2 decades. An increasing number of digital health tools, including wearable and smart devices, make early or real-time detection, monitoring, and intervention possible for CVD patients to prevent events with high morbidity and mortality. Model-based health economic analysis is a well-accepted tool used in the technology appraisal process by national institutes, such as the National Institute for Health and Care Excellence in the United Kingdom, to inform the clinical criteria and intervention cost for reasonable cost-effective use of health technology. In this review, the decision analytic model–based analyses evaluated the cost-effectiveness of DHIs and identified scenarios in which DHIs were likely to be accepted as cost-effective. The evidence generated by the health economic analyses facilitates the timely implementation of digital technologies in health care systems. Quality research is, therefore, highly warranted in health economic evaluation of DHIs for the management of chronic illnesses, in both developed and developing countries.

### Conclusions

This is the first systematic review of decision analytic model–based cost-effectiveness analyses of DHIs in the management of CVDs. Most of the identified analyses were published recently, and the majority were good quality cost-effectiveness analyses with an adequate duration of time frame. All included studies found the DHIs to be cost-effective.

## Appendix

Multimedia Appendix 1 Search terms.

Multimedia Appendix 2 CHEERS checklist and quality assessment of included studies.

## Abbreviations

CHD: coronary heart disease
CHEERS: Consolidated Health Economic Evaluation Reporting Standards
CoHD: congenital heart disease
CVD: cardiovascular disease
DHI: digital health intervention
FDA: Food and Drug Administration
HF: heart failure
HTA: health technology assessment
ICER: incremental cost-effectiveness ratio
NTS: nurse telephone support
QALY: quality-adjusted life year
SCA: sudden cardiac arrest
SMS: short message service
TEXT ME: Tobacco, Exercise and Diet Messages
TM: telemonitoring
WCD: wearable cardioverter-defibrillator
WTP: willingness-to-pay

## References

[1] Bhavnani SP, Narula J, Sengupta PP (2016). Mobile technology and the digitization of healthcare. Eur Heart J.

[2] (2018). US Food and Drug Administration.

[3] Noah B, Keller M, Mosadeghi S, Stein L, Johl S, Delshad S, Tashjian V, Lew D, Kwan J, Jusufagic A, Spiegel B (2018). Impact of remote patient monitoring on clinical outcomes: an updated meta-analysis of randomized controlled trials. NPJ Digital Medicine.

[4] (2011). World Health Orgnization.

[5] Harnett B (2006). Telemedicine systems and telecommunications. J Telemed Telecare.

[6] Tanawuttiwat T, Garisto JD, Salow A, Glad JM, Szymkiewicz S, Saltzman HE, Kutalek SP, Carrillo RG (2014). Protection from outpatient sudden cardiac death following ICD removal using a wearable cardioverter defibrillator. Pacing Clin Electrophysiol.

[7] (2018). World Health Organization.

[8] (2019). Champion Advocates Programme.

[9] Dendale P, De Keulenaer G, Troisfontaines P, Weytjens C, Mullens W, Elegeert I, Ector B, Houbrechts M, Willekens K, Hansen D (2012). Effect of a telemonitoring-facilitated collaboration between general practitioner and heart failure clinic on mortality and rehospitalization rates in severe heart failure: the TEMA-HF 1 (TElemonitoring in the MAnagement of Heart Failure) study. Eur J Heart Fail.

[10] Reid RD, Morrin LI, Beaton LJ, Papadakis S, Kocourek J, McDonnell L, Slovinec DME, Tulloch H, Suskin N, Unsworth K, Blanchard C, Pipe AL (2012). Randomized trial of an internet-based computer-tailored expert system for physical activity in patients with heart disease. Eur J Prev Cardiol.

[11] Widmer RJ, Collins NM, Collins CS, West CP, Lerman LO, Lerman A (2015). Digital health interventions for the prevention of cardiovascular disease: a systematic review and meta-analysis. Mayo Clin Proc.

[12] Neumann PJ, Sanders GD, Russell LB, Siegel JE, Ganiats TG (2016). Cost-Effectiveness in Health and Medicine Second Edition.

[13] (2018). World Health Organization.

[14] Michie S, Yardley L, West R, Patrick K, Greaves F (2017). Developing and evaluating digital interventions to promote behavior change in health and health care: recommendations resulting from an international workshop. J Med Internet Res.

[15] de la Torre-Diez I, López-Coronado M, Vaca C, Aguado JS, de Castro C (2015). Cost-utility and cost-effectiveness studies of telemedicine, electronic, and mobile health systems in the literature: a systematic review. Telemed J E Health.

[16] Husereau D, Drummond M, Petrou S, Carswell C, Moher D, Greenberg D, Augustovski F, Briggs AH, Mauskopf J, Loder E (2013). Consolidated Health Economic Evaluation Reporting Standards (CHEERS)--explanation and elaboration: a report of the ISPOR Health Economic Evaluation Publication Guidelines Good Reporting Practices Task Force. Value Health.

[17] Geng J, Yu H, Mao Y, Zhang P, Chen Y (2015). Cost effectiveness of dipeptidyl peptidase-4 inhibitors for type 2 diabetes. Pharmacoeconomics.

[18] Moher D, Liberati A, Tetzlaff J, Altman DG, PRISMA Group (2009). Preferred reporting items for systematic reviews and meta-analyses: the PRISMA statement. Ann Intern Med.

[19] Burn E, Nghiem S, Jan S, Redfern J, Rodgers A, Thiagalingam A, Graves N, Chow CK (2017). Cost-effectiveness of a text message programme for the prevention of recurrent cardiovascular events. Heart.

[20] Grustam AS, Severens JL, de Massari D, Buyukkaramikli N, Koymans R, Vrijhoef HJ (2018). Cost-effectiveness analysis in telehealth: a comparison between home telemonitoring, nurse telephone support, and usual care in chronic heart failure management. Value Health.

[21] Cano Martín JA, Martínez-Pérez B, de la Torre-Díez I, López-Coronado M (2014). Economic impact assessment from the use of a mobile app for the self-management of heart diseases by patients with heart failure in a Spanish region. J Med Syst.

[22] Mistry H, Gardiner HM (2013). The cost-effectiveness of prenatal detection for congenital heart disease using telemedicine screening. J Telemed Telecare.

[23] Whetten J, van der Goes DN, Tran H, Moffett M, Semper C, Yonas H (2018). Cost-effectiveness of Access to Critical Cerebral Emergency Support Services (ACCESS): a neuro-emergent telemedicine consultation program. J Med Econ.

[24] Nelson RE, Saltzman GM, Skalabrin EJ, Demaerschalk BM, Majersik JJ (2011). The cost-effectiveness of telestroke in the treatment of acute ischemic stroke. Neurology.

[25] Demaerschalk BM, Switzer JA, Xie J, Fan L, Villa KF, Wu EQ (2013). Cost utility of hub-and-spoke telestroke networks from societal perspective. Am J Manag Care.

[26] Nelson RE, Okon N, Lesko AC, Majersik JJ, Bhatt A, Baraban E (2016). The cost-effectiveness of telestroke in the Pacific Northwest region of the USA. J Telemed Telecare.

[27] Thokala P, Baalbaki H, Brennan A, Pandor A, Stevens JW, Gomersall T, Wang J, Bakhai A, Al-Mohammad A, Cleland J, Cowie MR, Wong R (2013). Telemonitoring after discharge from hospital with heart failure: cost-effectiveness modelling of alternative service designs. BMJ Open.

[28] Cowie MR, Simon M, Klein L, Thokala P (2017). The cost-effectiveness of real-time pulmonary artery pressure monitoring in heart failure patients: a European perspective. Eur J Heart Fail.

[29] Sandhu AT, Goldhaber-Fiebert JD, Owens DK, Turakhia MP, Kaiser DW, Heidenreich PA (2016). Cost-effectiveness of implantable pulmonary artery pressure monitoring in chronic heart failure. JACC Heart Fail.

[30] Schmier JK, Ong KL, Fonarow GC (2017). Cost-effectiveness of remote cardiac monitoring with the CardioMEMS heart failure system. Clin Cardiol.

[31] Martinson M, Bharmi R, Dalal N, Abraham WT, Adamson PB (2017). Pulmonary artery pressure-guided heart failure management: US cost-effectiveness analyses using the results of the CHAMPION clinical trial. Eur J Heart Fail.

[32] Healy CA, Carrillo RG (2015). Wearable cardioverter-defibrillator for prevention of sudden cardiac death after infected implantable cardioverter-defibrillator removal: a cost-effectiveness evaluation. Heart Rhythm.

[33] Grustam AS, Severens JL, van Nijnatten J, Koymans R, Vrijhoef HJ (2014). Cost-effectiveness of telehealth interventions for chronic heart failure patients: a literature review. Int J Technol Assess Health Care.

