# Supplementary material for: The Cost-Effectiveness of Digital Health Interventions on the Management of Cardiovascular Diseases: Systematic Review
Source: J Med Internet Res. 2019 Jun 17;21(6):e13166. doi: 10.2196/13166 (PMC6601257; doi:10.2196/13166)
Supplement: Multimedia Appendix 1 [file jmir_v21i6e13166_app1.pdf]

# Multimedia Appendix 1: Search Terms

We searched 8 electronic databases: Medline, Embase, CINAHL Complete, PsycINFO, Scopus, Web of Science, Center for Review and Dissemination, and Institute for IEEE Xplore, for relevant studies in November, 2018. The search strategy in each database is as follows.

<u>Medline</u>

1. exp cardiovascular diseases/
2. (heart failure or stroke or myocardial infarction or myocardial ischemia or (heart adj3 attack) or (heart adj3 disease) or (coronary adj3 disease) or angina pectories or (heart adj3 ischemia) or (heart adj3 revascularization) or (heart adj3 surgery) or (heart adj3 rehabilitation) or (cardiac adj3 rehabilitation) or atherosclerosis or brain vascular accident or cerebrovascular).ti,ab,hw,kw.
3. or/1-2
4. exp telemedicine/ or exp remote consultation/ or exp telemetry/ or exp home care services/ or exp telenursing/ or exp precision medicine/
5. exp Internet/ or exp cell phone/ or exp telephone/ or exp text messaging/ or exp electronic mail/ or exp mobile applications/
6. (digital health or mobile health or mhealth or m-health or electronic health or ehealth or e-health or health technology information).ti,ab,hw,kw.
7. (sensor* or wearable* or GPS or APP or robot*).ti,ab,hw,kw.
8. or/4-7
9. 3 and 8
10. exp models, economic/
11. exp economics/
12. (cost-utility or cost utility or cost-benefit or cost benefit or cost-effectiveness or cost effectiveness or cost consequence or cost-consequence or economic outcome or economic evaluation or economic impact or health economic* or economic modelling or economic assessment).ti,ab,hw,kw.
13. or/10-12
14. 9 and 13
15. limit 14 to clinical trial
16. limit 14 to review
17. or/15-16
18. 14 not 17
19. limit 18 to yr="2001-current"

<u>Embase</u>

1. exp cardiovascular disease/
2. (heart failure or stroke or myocardial infarction or myocardial ischemia or (heart adj3 attack) or (heart adj3 disease) or (coronary adj3 disease) or angina pectories or (heart adj3 ischemia) or (heart adj3 revascularization) or (heart adj3 surgery) or (heart adj3 rehabilitation) or (cardiac adj3 rehabilitation) or atherosclerosis or brain vascular accident or cerebrovascular).ti,ab,hw,kw.
3. or/1-2

4. exp telehealth/ or exp telemetry/ or exp rural health care/ or exp telecommunication/ or exp personalized medicine/
5. exp Internet/ or exp mobile phone/ or exp telephone/ or exp text messaging/ or exp e-mail/ or mobile application/
6. (digital health or mobile health or mhealth or m-health or electronic health or ehealth or e-health or health technology information).ti,ab,hw,kw.
7. (sensor* or wearable* or GPS or APP or robot*).ti,ab,hw,kw.
8. or/4-7
9. 3 and 8
10. exp economic model/
11. exp economics/
12. (cost-utility or cost utility or cost-benefit or cost benefit or cost-effectiveness or cost effectiveness or cost consequence or cost-consequence or economic outcome or economic evaluation or economic impact or health economic* or economic modelling or economic assessment).ti,ab,hw,kw.
13. or/10-12
14. 9 and 13
15. limit 14 to review
16. limit 14 to clinical trial
17. or/15-16
18. 14 not 17
19. limit 18 to yr="2001-current"

<u>CINAHL Complete</u>

| | | |
|---|---|---|
| S14 | S10 AND S13 | Search modes - Boolean/Phrase |
| S13 | S11 OR S12 | Search modes - Boolean/Phrase |
| S12 | cost-utility OR cost utility OR cost-benefit OR cost benefit OR cost-effectiveness OR cost effectiveness OR cost consequence OR cost-consequence OR economic outcome OR economic evaluation OR economic impact OR health economics OR economic modelling OR economic assessment | Search modes - Boolean/Phrase |
| S11 | (MH "Economics, Pharmaceutical") OR (MH "Costs and Cost Analysis+") | Search modes - Boolean/Phrase |
| S10 | S3 AND S9 | Search modes - Boolean/Phrase |
| S9 | S4 OR S5 OR S6 OR S7 OR S8 | Search modes - Boolean/Phrase |
| S8 | sensor OR wearable OR GPS OR APP OR robot | Search modes - Boolean/Phrase |
| S7 | digital health OR mobile health OR mhealth OR m-health OR electronic health OR ehealth OR e-health OR health technology information | Search modes - Boolean/Phrase |
| S6 | (MH "Home Health Care+") | Search modes - Boolean/Phrase |
| S5 | (MH "Telemetry") | Search modes - Boolean/Phrase |
| S4 | (MH "Internet+") OR (MH "Telehealth+") OR (MH "Telephone+") | Search modes - Boolean/Phrase |
| S3 | S1 OR S2 | Search modes - Boolean/Phrase |

| | | |
|---|---|---|
| S2 | heart failure OR stroke OR myocardial infarction OR myocardial ischemia OR heart attack OR heart disease OR coronary disease OR angina pectories OR heart ischemia OR heart revascularization OR heart surgery OR heart rehabilitation OR cardiac rehabilitation OR atherosclerosis OR brain vascular accident OR cerebrovascular | Search modes - Boolean/Phrase |
| S1 | (MH "Cardiovascular Diseases+") | Search modes - Boolean/Phrase |

<u>PsycINFO</u>

1. exp Cardiovascular Disorders/
2. (heart failure or stroke or myocardial infarction or myocardial ischemia or (heart adj3 attack) or (heart adj3 disease) or (coronary adj3 disease) or angina pectories or (heart adj3 ischemia) or (heart adj3 revascularization) or (heart adj3 surgery) or (heart adj3 rehabilitation) or (cardiac adj3 rehabilitation) or atherosclerosis or brain vascular accident or cerebrovascular).mp.
3. or/1-2
4. exp telemedicine/ or exp telemetry/ or exp home care/
5. exp INTERNET/ or exp TELEPHONE SYSTEMS/ or exp Text Messaging/ or exp Computer mediated communication/ or exp Mobile Devices/
6. (digital health or mobile health or mhealth or m-health or electronic health or ehealth or e-health or health technology information).mp.
7. (sensor* or wearable* or GPS or APP or robot*).mp.
8. or/4-7
9. 3 and 8
10. exp economics/
11. (cost-utility or cost utility or cost-benefit or cost benefit or cost-effectiveness or cost effectiveness or cost consequence or cost-consequence or economic outcome or economic evaluation or economic impact or health economic* or economic modelling or economic assessment).mp.
12. or/10-11
13. 9 and 12

<u>Scopus</u>

(TITLE-ABS-KEY (cardiovascular AND disease ) AND TITLE-ABS-KEY (telehealth OR telemedicine OR telenursing OR telemetry OR (rural AND health AND care) OR (remote AND consultation) OR (home AND care AND service) OR (personalised AND medicine) OR (digital AND health) OR (mobile AND health) OR mhealth OR m-health) OR TITLE-ABS-KEY ((electronic AND health) OR ehealth OR e-health OR (health AND technology AND information)) AND TITLE-ABS-KEY (cost-utility OR (cost AND utility ) OR cost-benefit OR (cost AND benefit) OR cost-effectiveness OR (cost AND effectiveness) OR (cost AND consequence) OR cost-consequence) OR TITLE-ABS-KEY ((economic AND outcome) OR (economic AND evaluation) OR (economic AND impact) OR (economic AND modelling) OR(economic AND assessment)))

<u>Web of Science</u>

| | |
|---|---|
| # 4 | #3 AND #2 AND #1 |
| # 3 | TS= (cost-utility OR (cost utility) OR cost-benefit OR (cost benefit) OR cost-effectiveness OR (cost effectiveness) OR (cost consequence) OR cost-consequence OR (economic outcome) OR (economic evaluation) OR (economic impact) OR (economic modelling) OR (economic assessment)) |
| # 2 | TS= (telehealth OR telemedicine OR telenursing OR telemetry OR (rural health care) OR (remote consultation) OR (home care service) OR (personalised medicine) OR (digital health) OR (mobile health) OR mhealth OR m-health OR (electronic health) OR ehealth OR e-health OR (health technology information)) |
| # 1 | cardiovascular disease |

<u>Center for Review and Dissemination</u>

(cardiovascular disease OR heart disease OR vascular disease) AND (telehealth OR telemedicine OR telenursing OR telemetry OR (rural health care) OR (remote consultation) OR (home care service) OR (personalised medicine) OR (digital health) OR (mobile health) OR mhealth OR m-health OR (electronic health) OR ehealth OR e-health OR (health technology information)) AND (cost-utility OR (cost utility) OR cost-benefit OR (cost benefit) OR cost-effectiveness OR (cost effectiveness) OR (cost consequence) OR cost-consequence OR (economic outcome) OR (economic evaluation) OR (economic impact) OR (economic modelling) OR (economic assessment))

<u>Institute for IEEE Xplore</u>

(((cardiovascular AND disease) AND (telehealth OR telemedicine OR telenursing OR telemetry OR (rural AND health AND care) OR (remote AND consultation) OR (home AND care AND service) OR (personalised AND medicine) OR (digital AND health) OR (mobile AND health) OR mhealth OR m-health) OR (electronic AND health) OR ehealth OR e-health OR (health AND technology AND information))) AND (cost-utility OR (cost AND utility) OR cost-benefit OR (cost AND benefit) OR cost-effectiveness OR (cost AND effectiveness) OR (cost AND consequence) OR cost-consequence) OR (economic AND outcome) OR (economic AND evaluation) OR (economic AND impact) OR (economic AND modelling) OR (economic AND assessment))
