# Supplementary material for: The Cost-Effectiveness of Digital Health Interventions on the Management of Cardiovascular Diseases: Systematic Review
Source: J Med Internet Res. 2019 Jun 17;21(6):e13166. doi: 10.2196/13166 (PMC6601257; doi:10.2196/13166)
Supplement: Multimedia Appendix 2 [file jmir_v21i6e13166_app2.pdf]

Multimedia Appendix 2: CHEERS checklist and quality assessment of included studies

Table of contents

CHEERS checklist 1
Quality assessment of included studies 3

CHEERS checklist

Item No	Item	Recommendation
1	Title	Identify the study as an economic evaluation or use more specific terms such as "cost-effectiveness analysis", and describe the interventions compared.
2	Abstract	Provide a structured summary of objectives, perspective, setting, methods (including study design and inputs), results (including base case and uncertainty analyses), and conclusions.
3	Background and Objectives	Provide an explicit statement of the broader context for the study. Present the study question and its relevance for health policy or practice decisions.
4	Target Population & subgroups	Describe characteristics of the base case population and subgroups analysed, including why they were chosen.
5	Setting & Location	State relevant aspects of the system(s) in which the decision(s) need(s) to be made.
6	Study Perspective	Describe the perspective of the study and relate this to the costs being evaluated.
7	Comparators	Describe the interventions or strategies being compared and state why they were chosen.
8	Time Horizon	State the time horizon(s) over which costs and consequences are being evaluated and say why appropriate.
9	Discount Rate	Report the choice of discount rate(s) used for costs and outcomes and say why appropriate.
10	Choice of Health Outcomes	Describe what outcomes were used as the measure(s) of benefit in the evaluation and their relevance for the type of analysis performed.
11	Measurement of Effectiveness	Single study-based estimates: Describe fully the design features of the single effectiveness study and why the single study was a sufficient source of clinical effectiveness data.<br>Synthesis-based estimates: Describe fully the methods used for identification of included studies and synthesis of clinical effectiveness data.
12	Measurement & Valuation of Preference-based Outcomes	If applicable, describe the population and methods used to elicit preferences for outcomes.
13	Estimating Resources & Costs	Single study-based economic evaluation: Describe approaches used to estimate resource use

		associated with the alternative interventions. Describe primary or secondary research methods for valuing each resource item in terms of its unit cost. Describe any adjustments made to approximate to opportunity costs.<br>Model-based economic evaluation: Describe approaches and data sources used to estimate resource use associated with model health states. Describe primary or secondary research methods for valuing each resource item in terms of its unit cost. Describe any adjustments made to approximate to opportunity costs.
14	Currency, Price Date & Conversion	Report the dates of the estimated resource quantities and unit costs. Describe methods for adjusting estimated unit costs to the year of reported costs if necessary. Describe methods for converting costs into a common currency base and the exchange rate.
15	Choice of Model	Describe and give reasons for the specific type of decision analytical model used. Providing a figure to show model structure is strongly recommended.
16	Assumptions	Describe all structural or other assumptions underpinning the decision-analytical model.
17	Analytic Methods	Describe all analytical methods supporting the evaluation. This could include methods for dealing with skewed, missing, or censored data; extrapolation methods; methods for pooling data; approaches to validate or make adjustments (such as half cycle corrections) to a model; and methods for handling population heterogeneity and uncertainty.
18	Study Parameters	Report the values, ranges, references, and, if used, probability distributions for all parameters. Report reasons or sources for distributions used to represent uncertainty where appropriate. Providing a table to show the input values is strongly recommended.
19	Incremental Costs & Outcomes	For each intervention, report mean values for the main categories of estimated costs and outcomes of interest, as well as mean differences between the comparator groups. If applicable, report incremental cost-effectiveness ratios.
20	Characterizing Uncertainty	Single study-based economic evaluation: Describe the effects of sampling uncertainty for the estimated incremental cost and incremental effectiveness parameters, together with the impact of methodological assumptions (such as discount rate, study perspective).<br>Model-based economic evaluation: Describe the effects on the results of uncertainty for all input

		parameters, and uncertainty related to the structure of the model and assumptions.
21	Characterizing Heterogeneity	If applicable, report differences in costs, outcomes, or cost effectiveness that can be explained by variations between subgroups of patients with different baseline characteristics or other observed variability in effects that are not reducible by more information.
22	Study Findings, Limitations, Generalizability & Current Knowledge	Summarise key study findings and describe how they support the conclusions reached. Discuss limitations and the generalisability of the findings and how the findings fit with current knowledge.
23	Source of Funding	Describe how the study was funded and the role of the funder in the identification, design, conduct, and reporting of the analysis. Describe other non-monetary sources of support.
24	Conflicts of Interest	Describe any potential for conflict of interest of study contributors in accordance with journal policy. In the absence of a journal policy, we recommend authors comply with International Committee of Medical Journal Editors recommendations.

Quality assessment of included studies
(Ordered according to the results)

Burn *et al.* [1]
Total Quality Score: 22/24

Item	Reported Data	Quality Score (0-1)
**Title & Abstract**		
Title	Cost-effectiveness of a text message programme for the prevention of recurrent cardiovascular events	1
Abstract	The structured abstract with objective, methods, results, conclusions and trial registration number was used. But the perspective and setting were not clearly specified.	0
**Introduction**		
Background and Objectives	Context and study questions were clear.	1
**Methods**		
Target Population & subgroups	Patients admitted to Australia public hospital, with prior myocardial infarction, coronary artery bypass graft surgery, percutaneous coronary intervention or 50% or greater stenosis in at least one major epicardial vessel on coronary angiography, were considered. The ages for female and male patients were 57 and 58.	1
Setting & Location	Australia pubic hospital	1
Study Perspective	Health system perspective	1
Comparators	With Tobacco, Exercise and Diet Messages (TEXT ME) versus without TEXT ME	1
Time Horizon	Lifetime	1

Discount Rate	Annual discount rate of 3%	1
Choice of Health Outcomes	Quality adjusted-life years (QALYs), numbers of myocardial infarction, and strokes	1
Measurement of Effectiveness	The effectiveness was retrieved from literature reviews, including the results from a randomized controlled trial and four meta-analysis.	1
Measurement & Valuation of Preference-based Outcomes	The baseline utility and the utility of 6-month text messages strategy were estimated from a health survey conducted in TEXT ME trial. The utility associated with a myocardial infarction event and stroke were retrieved from the literature review with referencing.	1
Estimating Resources & Costs	Primary care costs were based on records from the Medicare Benefits Schedule and the Pharmaceutical Benefits Scheme. Hospital costs relating to major vascular events were based on the age- and gender-specific cost per hospital separation reported for Australia. The costs of the intervention were estimated in consultation with the programme staff.	1
Currency, Price Date & Conversion	2014 AUD	1
Choice of Model	Markov model	1
Assumptions	The key simplifying assumption was that individuals could only have one of either an of myocardial infarction or a stroke, after which they moved to and then remained in the history of secondary event state until death.	1
Analytic Methods	Probabilistic sensitivity analysis with 1,000 Monte Carlo simulations was conducted and five scenario analysis were considered.	1
**Results**		
Study Parameters	Input values and ranges were specified.	1
Incremental Costs & Outcomes	TEXT ME was expected to lead to 563 fewer myocardial infarctions, 361 fewer strokes and 1143 additional QALYs, with an overall saving of $10.56 million for the health system over the patients' lifetimes.	1
Characterizing Uncertainty	Parameter uncertainty had little effect on the conclusion that TEXT ME was cost-effective, which was shown in a cost-effectiveness plane. TEXT ME was cost-saving in all the individual scenario analysis, whilst it was cost-effective when all scenario run simultaneously.	1
Characterizing Heterogeneity	Potential heterogeneity of patients was not addressed.	0
**Discussion**		
Study Findings, Limitations, Generalizability & Current Knowledge	Findings, limitations, generalizability, and current knowledge were discussed.	1
**Other**		
Source of Funding	BUPA Foundation	1
Conflicts of Interest	The authors declared no conflicts of interest.	1

Grustam *et al.* [2]

Total Quality Score: 22/24

Item	Reported Data	Quality Score

		(0-1)
**Title & Abstract**		
Title	Cost-Effectiveness analysis in telehealth: a comparison between home telemonitoring, nurse telephone support, and usual care in chronic heart failure management	1
Abstract	The structured abstract with objective, methods, results, and conclusions was used. But the setting was not specified.	0
**Introduction**		
Background and Objectives	Context and study questions were clear.	1
**Methods**		
Target Population & subgroups	Patients with chronic heart failure aged 70 years and older, in all New York Heart Association (NYHA) classes of severity, were considered.	1
Setting & Location	Netherlands	1
Study Perspective	The third-party payer's perspective	1
Comparators	Home telemonitoring, nurse telephone support, and usual care	1
Time Horizon	20 years	1
Discount Rate	The costs and effects were discounted by a 4% and 1.5% yearly rate, respectively.	1
Choice of Health Outcomes	life expectancy and QALYs	1
Measurement of Effectiveness	The data on effectiveness was estimated from a manufacturer database.	1
Measurement & Valuation of Preference-based Outcomes	The utility values for each NYHA class were retrieved from a manufacturer database and constructed using the Dutch utility weights.	1
Estimating Resources & Costs	The personnel- and hospital-related costs were from the Dutch health care costing manual. And the costs of the telemonitoring system were acquired from the manufacturer and adjusted in accordance with the market research.	1
Currency, Price Date & Conversion	2015 EUR, with conversion based on the consumer price index	1
Choice of Model	Markov model	1
Assumptions	(1)20 years was assumed to be sufficient to analyze the benefits of the interventions, and could be considered a life time horizon. (2) The transition probabilities measured in a limited time frame of 240 to 450 days would continue unaltered for 20 years. (3) The hospitalization costs were assumed to be treatment arm–independent, but NYHA class–dependent. (4) The utility values were assumed to connect to the severity of the disease.	1
Analytic Methods	Probabilistic sensitivity analysis, threshold analysis, subgroup analysis, and scenario analysis were conducted.	1
**Results**		
Study Parameters	Input values and ranges were specified.	1
Incremental Costs & Outcomes	The incremental cost-effectiveness ratios (ICERs) were €12,479 (home telemonitoring versus usual care), €8,270 (for nurse telephone support versus usual care), and -€23,661 (for home telemonitoring versus nurse telephone support).	1
Characterizing Uncertainty	The scenario including telenurse cost inputs in nurse telephone support yielded results that were slightly different from those from the scenario excluding this cost, when comparing all NYHA classes of severity.	1

	The probabilistic sensitivity analysis suggested that there was a very low probability of home telemonitoring being cost-effective when nurse telephone support was available for the management of patients with chronic heart failure.	
Characterizing Heterogeneity	Nurse telephone support dominated home telemonitoring, compared with usual care, in all NYHA classes except NYHA IV.	1
**Discussion**		
Study Findings, Limitations, Generalizability & Current Knowledge	Findings, limitations, generalizability, and current knowledge were discussed.	1
**Other**		
Source of Funding	The funding was not specified.	0
Conflicts of Interest	Source of financial support was reported.	1

<u>Martín *et al.*</u> [3]

Total Quality Score:12 /24

Item	Reported Data	Quality Score (0-1)
**Title & Abstract**		
Title	Economic impact assessment from the use of a mobile app for the self-management of heart diseases by patients with heart failure in a Spanish region	1
Abstract	No structured abstract was used.	0
**Introduction**		
Background and Objectives	Context and study questions were clear.	1
**Methods**		
Target Population & subgroups	Heart failure (HF) patients were considered. But the age and severity were not specified.	0
Setting & Location	Castile and Leon	1
Study Perspective	No clear perspective was specified in the method.	0
Comparators	With the app versus without the app	1
Time Horizon	Not clear.	0
Discount Rate	The discount rate was not specified in the method.	0
Choice of Health Outcomes	QALYs	1
Measurement of Effectiveness	Authors mentioned the inputs were retrieved from literature reviews and statistics center. But it was unclear from which study each parameter estimate was obtained (each parameter was not referenced, respectively).	0
Measurement & Valuation of Preference-based Outcomes	The measurement of preference-based outcomes was not specified.	0
Estimating Resources & Costs	The costs of the tool worth 100 euros. The healthcare cost and non-sanitary cost were retrieved from the Ministry of Health, Social Policy and Equality.	1
Currency, Price Date & Conversion	2011 EUR	1
Choice of Model	Markov model	1
Assumptions	The assumption was not clearly specified.	0
Analytic Methods	A univariate sensitivity analysis was carried out to assess the robustness of the results.	1
**Results**		
Study Parameters	Study parameters and the ranges were not specified.	0
Incremental Costs & Outcomes	The cost for introduction of the app was €19.012 per patient and that for no introduction was €28.315. The ICER was €9.303/QALY. However, the health outcome was not clearly specified and how to calculate the ICER was not clear.	0

Characterizing Uncertainty	The results of a univariate analysis were not presented.	0
Characterizing Heterogeneity	Potential heterogeneity of HF patients was not specified.	0
**Discussion**		
Study Findings, Limitations, Generalizability & Current Knowledge	Findings, limitations, generalizability, and current knowledge were discussed.	1
**Other**		
Source of Funding	Ministerio de Economía y Competitividad, Spain.	1
Conflicts of Interest	The authors declared that they have no conflict of interest.	1

Mistry *et al.* [4]

Total Quality Score: 17/24

Item	Reported Data	Quality Score (0-1)
**Title & Abstract**		
Title	The cost-effectiveness of prenatal detection for congenital heart disease using telemedicine screening	1
Abstract	No structured abstract was used.	0
**Introduction**		
Background and Objectives	Context and study questions were clear.	1
**Methods**		
Target Population & subgroups	Standard-risk pregnant women classified into 4 subgroups were considered.	1
Setting & Location	UK	1
Study Perspective	UK healthcare perspective	1
Comparators	With congenital heart disease screening versus without congenital heart disease	1
Time Horizon	Lifetime	1
Discount Rate	Annual discount rate of 3.5%	1
Choice of Health Outcomes	QALYs	1
Measurement of Effectiveness	The measurement of effectiveness was not specified.	0
Measurement & Valuation of Preference-based Outcomes	The mother's utility was retrieved from a study with referencing but the utility of the newborn children was not clearly specified.	0
Estimating Resources & Costs	The estimated resources were presented but not clearly specified.	0
Currency, Price Date & Conversion	The currency, price date and conversion were not clearly specified.	0
Choice of Model	Decision tree model	1
Assumptions	(1) The authors assumed that telemedicine had a 97% sensitivity and 96% specificity rate. (2) It was assumed that a further pregnancy with a normal outcome would occur, with a delay of one year in about 50% of women. (3) All 'standard-risk' women screened over 15 months could be reviewed in 11 working weeks. (4) It was assumed that each patient in the model would incur a cost to the health service. (5) It was assumed that on average each child would live to their expected lifetime.	1
Analytic Methods	Bootstrapping was used to stabilize the mean and to generate 95% confidence intervals around the mean value for the skewed cost and effectiveness data. One-way and probabilistic sensitivity analysis were also conducted.	1
**Results**		

Study Parameters	Input values and ranges were specified.	1
Incremental Costs & Outcomes	In the base-case assumption, the arm that all women received telemedicine screening was dominant.	1
Characterizing Uncertainty	The ICER of one-way sensitivity analysis were clearly presented. The probability of a screening strategy with telemedicine being cost-effective was nearly 100% at a willingness-to-pay (WTP) of £20,000 per QALY.	1
Characterizing Heterogeneity	Heterogeneity of patients was not addressed.	0
**Discussion**		
Study Findings, Limitations, Generalizability & Current Knowledge	Findings, limitations, generalizability, and current knowledge were discussed.	1
**Other**		
Source of Funding	The present study received no funding.	1
Conflicts of Interest	The conflicts of interest were not declared.	0

Whetten *et al.* [5]

Total Quality Score: 18/24

Item	Reported Data	Quality Score (0-1)
**Title & Abstract**		
Title	Cost-effectiveness of access to critical cerebral emergency support services (ACCESS): a neuro-emergent telemedicine consultation program	1
Abstract	The structured abstract with aims, methods, results, and conclusion was used, providing complete summary of the study.	1
**Introduction**		
Background and Objectives	Context and study questions were clear.	1
**Methods**		
Target Population & subgroups	The target population was not clearly specified.	0
Setting & Location	New Mexico	1
Study Perspective	The study perspective was not clearly specified in the method.	0
Comparators	With the program versus without the program	1
Time Horizon	90 days	1
Discount Rate	Discounting was not reported as 0% as guideline suggested.	0
Choice of Health Outcomes	QALYs	1
Measurement of Effectiveness	The effectiveness data was estimated from the ACCESS program, without clear description of the trial.	0
Measurement & Valuation of Preference-based Outcomes	The utility values were retrieved from literature reviews with clear referencing.	1
Estimating Resources & Costs	The potential cost of care included ACCESS consultation fee, transfer costs, cost of tissue plasminogen activator administration, and final diagnosis costs. Final diagnosis costs were taken from inpatient costs, length of average stay, and other medical costs associated with 90-day stroke outcomes. The costs were presented in a table with individual referencing.	1
Currency, Price Date & Conversion	2015 US dollars, using the medical care services component of the Consumer Price Index	1
Choice of Model	Decision tree model	1
Assumptions	The assumptions were not clearly specified.	0

Analytic Methods	One-way sensitivity analysis and probabilistic analysis were completed.	1
**Results**		
Study Parameters	Input values and ranges were specified.	1
Incremental Costs & Outcomes	The use of ACCESS had the potential to save $4,241 ($3,952–$4,438) per patient and increase QALYs by 0.20 (0.14–0.22).	1
Characterizing Uncertainty	The large swings in the parameters indicated the ACCESS program was still cost-saving. The Monte Carlo simulations show potential mean cost savings per patient of $4,197 ($3,952–$4,438). The mean QALYs per patient was 0.18 (0.14–0.22).	1
Characterizing Heterogeneity	Heterogeneity of patients was not addressed	0
**Discussion**		
Study Findings, Limitations, Generalizability & Current Knowledge	Findings, limitations, generalizability, and current knowledge were discussed.	1
**Other**		
Source of Funding	Centers for Medicare & Medicaid Services	1
Conflicts of Interest	The authors are employees of the University of New Mexico. Peer reviewers on this manuscript have received an honorarium from JME for their review work, but have no other relevant financial relationships to disclose.	1

Nelson *et al.* [6]

Total Quality Score: 22/24

Item	Reported Data	Quality Score (0-1)
**Title & Abstract**		
Title	The cost-effectiveness of telestroke in the treatment of acute ischemic stroke	1
Abstract	A structured abstract with objectives, methods, results, and conclusion was used, providing complete summary of the study.	1
**Introduction**		
Background and Objectives	Context and study questions were clear.	1
**Methods**		
Target Population & subgroups	Patients with acute ischemic stroke at a spoke hospital were considered. But the age and severity were not specified.	0
Setting & Location	Hub-and-spoke telestroke system	1
Study Perspective	A societal perspective.	1
Comparators	Telestroke versus usual care	1
Time Horizon	Both the 90-day and lifetime timeframes	1
Discount Rate	Annual discount rate of 3%.	1
Choice of Health Outcomes	QALYs	1
Measurement of Effectiveness	The effectiveness data was estimated from the literatures and the Stroke Telemedicine for Arizona Rural Residents telestroke network.	1
Measurement & Valuation of Preference-based Outcomes	The utility weight associated with mRS scores used was obtained from a previous study and had been used in several other cost-effectiveness analyses of stroke.	1
Estimating Resources & Costs	Telestroke infrastructure costs for both spoke and hub facilities included equipment, staffing, and training and were taken from the Utah Telehealth Network and STARR experiences. Patient care costs	1

	were obtained from published literature and included tissue plasminogen activator and transfer costs (which were independent of stroke severity), as well as hospital, rehabilitation, skilled nursing facility, and daily caregiver costs.	
Currency, Price Date & Conversion	2008 US dollars	1
Choice of Model	Type of decision-analytic model was not specified. But a figure of the model was provided.	1
Assumptions	(1) It's assumed a telestroke system with 8 spokes (range 6–12), each of which had 12 telestroke consults per year (range 6–30), and 1 hub with 4 neurologists (range 3–5) rotating telestroke calls from either the office/hospital (1 shared hospital-based telestroke unit) or the home (each with a home telestroke unit). (2) Patients with an modified Rankin Score (mRS) score (a 6-point disability scale) of 0 were assumed to be discharged to home, while those with scores between 1 and 5 could be discharged to home, a rehabilitation facility, or a nursing home. (3) In patients who were discharged to rehabilitation, the score was assumed to improve by 1 point at 90 days; the initial mRS score was assumed to not change in patients who were discharged either to home or to a skilled nursing facility.	1
Analytic Methods	One-way sensitivity analysis and probabilistic sensitivity analysis were conducted.	1
**Results**		
Study Parameters	Input values were specified.	1
Incremental Costs & Outcomes	Telestroke resulted in an ICER of $108,363/QALY in the 90-day horizon and $2,449/QALY in the lifetime horizon.	1
Characterizing Uncertainty	The 90-day horizon models were sensitive to number of patients per spoke and the cost of transfer while the life-time models were sensitive to the probability of mRS score of 5 and annual medical cost. For the 90-day and lifetime horizons, 37.5% and 99.7% of 10,000 Monte Carlo simulations yielded ICERs $50,000/QALY.	1
Characterizing Heterogeneity	Heterogeneity of patients was not addressed.	0
**Discussion**		
Study Findings, Limitations, Generalizability & Current Knowledge	Findings, limitations, generalizability, and current knowledge were discussed.	1
**Other**		
Source of Funding	National Institute of Health	1
Conflicts of Interest	The author disclosed the financial and non-financial association with the commercial, academic, and other entities pertinent to the manuscript.	1

Demaerschalk *et al.* [7]

Total Quality Score:19 /24

Item	Reported Data	Quality Score (0-1)
**Title & Abstract**		
Title	Cost utility of hub-and-spoke telestroke networks from societal perspective	1
Abstract	A structured abstract with background, objectives, study design and method, results, and conclusion was used. But the setting was not provided.	0

**Introduction**		
Background and Objectives	Context and study questions were clear.	1
**Methods**		
Target Population & subgroups	Acute ischemic stroke patients with a mean age of 68 were considered. But the severity of the patients was not specified.	0
Setting & Location	The setting and location were not clearly specified.	0
Study Perspective	The study perspective was not clearly specified in the method.	0
Comparators	Telestroke versus no telestroke	1
Time Horizon	Lifetime	1
Discount Rate	Annual discount rate of 3%	1
Choice of Health Outcomes	QALYs	1
Measurement of Effectiveness	Data on mRS at 3 months for IV thrombolysis with different onset-to-treatment times and endovascular stroke therapies were obtained from clinical trials, and were adjusted based on the assumption that mortality was the same for patients with versus without IV thrombolysis and for those with endovascular stroke therapy versus without endovascular stroke therapy. The data was displayed in a table with individual referencing.	1
Measurement & Valuation of Preference-based Outcomes	The utility values were retrieved from the literatures and were referenced individually.	1
Estimating Resources & Costs	Costs included the following components: (1) Telestroke setup and maintenance costs, (2) initial hospitalization costs, (3) post-acute stroke care costs (including rehabilitation and nursing home costs), and (4) caregiver costs, which were obtained from the literature and publicly available data, with individual referencing.	1
Currency, Price Date & Conversion	2011 US dollars	1
Choice of Model	Markov model	1
Assumptions	(1) Acute ischemic stroke patients could only transfer from a less severe to a more severe health state or remain in the same health state at each cycle. (2) Stroke treatments between a telestroke network and no network differed only during the initial hospitalization for acute ischemic stroke, not after discharge from acute care. (3) Incremental effectiveness associated with treatments in a telestroke network only resulted from IV thrombolysis or endovascular stroke therapy during the initial hospitalization for the first-time acute ischemic stroke. (4) There was no difference in stroke-related mortality between patients with and without IV thrombolysis, and between patients with and without endovascular stroke therapy during hospitalization. (5) Rate of recurrent stroke was the same regardless of the treatment received during the initial hospitalization for acute ischemic stroke.	1
Analytic Methods	One-way and two-way sensitivity analysis were conducted.	1
**Results**		
Study Parameters	The study parameters were specified.	1
Incremental Costs & Outcomes	Patients treated in a telestroke network incurred $1436 lower costs and gained 0.02 QALYs over a lifetime.	1

Characterizing Uncertainty	The one-way sensitivity analyses showed that the results were robust, with a telestroke network being the dominant strategy in all scenarios except when the spoke-to-hub transfer rate was varied. When varying the transfer rate from 0% to 100%, the model showed that the network remained a dominant strategy when the transfer rate increased to 60% and remained cost-effective with a WTP threshold of $50,000 per QALY when the transfer rate increased to 90%.	1
Characterizing Heterogeneity	Heterogeneity of patients was not addressed.	0
**Discussion**		
Study Findings, Limitations, Generalizability & Current Knowledge	Findings, limitations, generalizability, and current knowledge were discussed.	1
**Other**		
Source of Funding	Genentech, Inc	1
Conflicts of Interest	The authors reported the disclosure.	1

Nelson *et al*. [8]

Total Quality Score: 19/24

Item	Reported Data	Quality Score (0-1)
**Title & Abstract**		
Title	The cost-effectiveness of telestroke in the Pacific Northwest region of the USA	1
Abstract	A structured abstract with introduction, methods, results, and conclusion are used. However, the setting and the input in the model were not clearly specified.	0
**Introduction**		
Background and Objectives	Context and study questions were clear.	1
**Methods**		
Target Population & subgroups	Acute ischemic stroke patients presenting to a spoke hospital within 4.5 h were considered. But the age and severity were not specified.	0
Setting & Location	The setting and the location were not specified in the method.	0
Study Perspective	The perspective of both the hub and the spoke	1
Comparators	Telestroke versus no telestroke	1
Time Horizon	A patient's inpatient stay	1
Discount Rate	The authors reported no discount was applied.	1
Choice of Health Outcomes	QALYs	1
Measurement of Effectiveness	The effectiveness data was from a single trial without clear description of the trial.	0
Measurement & Valuation of Preference-based Outcomes	The utility values were estimated from literatures, with individual referencing.	1
Estimating Resources & Costs	The cost and reimbursement data were retrieved from encounter-level financial dataset. Medicare Severity Diagnosis-Related Groups 61-66 were also used. This data included emergency department, rehabilitation, and treatment for acute ischemic stroke with or without telestroke. The fixed cost of telestroke was retrieved from the local telestroke network.	1
Currency, Price Date & Conversion	2013US dollars	1
Choice of Model	Type of decision-analytic model was not clearly specified. However, a figure was shown.	1
Assumptions	(1) The model assumed that after presenting at the emergency department of this hospital, the patient	1

	could receive tissue plasminogen activator or not. (2) Spoke facilities were assumed to be responsible for 0%, 50%, and 100% of implementation expenses. (3) The mRS scores based on discharge location were assumed as followed: home = 0–1, rehabilitation = 2–3, skilled nursing facility = 4–5, and death = 6.	
Analytic Methods	One-way sensitivity analysis, probabilistic sensitivity analysis, and scenario analysis were conducted. Subgroup analysis for each National Institute of Health Stroke Scale severity category was also complete.	1
**Results**		
Study Parameters	Model inputs and ranges were adequately described.	1
Incremental Costs & Outcomes	From the spoke perspective, telestroke had ICERs of US$1322/QALY, US$25,991/QALY and US$50,687/QALY when responsible for 0%, 50%, and 100% of these costs, respectively. From the hub perspective, telestroke had ICERs of US$71,703/QALY, US$47,033/QALY, and US$22,363/QALY, respectively.	1
Characterizing Uncertainty	The results of probabilistic analysis were shown but the results of one-way sensitivity analysis were not clearly specified.	0
Characterizing Heterogeneity	When analyzed by severity subgroups, telestroke was most cost-effective compared to non-telestroke assisted care from the spoke perspective for severe stroke patients with ICERs ranging from US$7794/QALY to US$40,071/QALY. The overall ICER from the hub perspective ranged from US$22,363/ QALY to US$71,703/QALY with the lowest ICERs, again, seen in the severe stroke patients.	1
**Discussion**		
Study Findings, Limitations, Generalizability & Current Knowledge	Findings, limitations, generalizability, and current knowledge were discussed.	1
**Other**		
Source of Funding	US Health Resources and Services Administration, the Office for the Advancement of Telehealth, Health Resources and Services Administration, Department of Health and Human Services, the Department of Veterans Affairs, Veterans Health Administration, Office of Research and Development, Health Services Research and Development Service	1
Conflicts of Interest	The author(s) declared no potential conflicts of interest with respect to the research, authorship, and/or publication of this article.	1

Thokala *et al.* [9]

Total Quality Score: 21/24

Item	Reported Data	Quality Score (0-1)
**Title & Abstract**		
Title	Telemonitoring after discharge from hospital with heart failure: cost-effectiveness modelling of alternative service designs	1
Abstract	Structured summary of objectives, design, setting, patients, interventions, main outcome measures, and	0

	results was used. However, the perspective was not specified.	
**Introduction**		
Background and Objectives	Context and study questions were clear.	1
**Methods**		
Target Population & subgroups	Only patients with HF were considered. Patients' age and severity of HF were not specified.	0
Setting & Location	Acute hospital in the UK	1
Study Perspective	The National Health Service in England and Wales	1
Comparators	Usual care, structured telephone support with human-to-human contact, structured telephone support with human-to-machine interface, and telemonitoring	1
Time Horizon	30 years	1
Discount Rate	Annual discount rate of 3.5%	1
Choice of Health Outcomes	QALYs	1
Measurement of Effectiveness	(1) The baseline monthly mortality rate was retrieved from Assessment of Reduction in Mortality and morbidity (CHARM) study. (2) Mean numbers of HF-related and all-cause hospitalization were estimated from a meta-analysis. (3) Hazard ratios for all-cause mortality, all-cause hospitalizations and HF-related hospitalizations for the different interventions were estimated from a meta-analysis.	1
Measurement & Valuation of Preference-based Outcomes	The utility for discharged HF patients was from literature reviews., with detailed approach for data retrieval.	1
Estimating Resources & Costs	Costs of interventions other than usual care were broken down into the costs of the device, monitoring cost, and medical care cost and were estimated using bottom-up costing methods for the National Health Service Foundation trusts for 250 HF patients. The inpatient admission cost for hospitalization was from National Health Service references costs.	1
Currency, Price Date & Conversion	2011 GBP	1
Choice of Model	Markov model	1
Assumptions	(1) The interventions were provided for the six months following discharge from the hospital. (2) Patients were provided usual care as description in The Trans-European Network-Home-Care Managments System (TEN-HMS) study 6 months later. (3) Treatment effectiveness and cost only lasted for 6 months. (4) Any HF-related rehospitalization was assumed to result in a disutility of 0.1 and last for 1 year. (5) The cost of hospitalization for cause other than HF was assumed to the mean cost of admission for general population.	1
Analytic Methods	Scenario analyses, threshold analyses, and probabilistic analysis were considered.	1
**Results**		
Study Parameters	Model inputs were adequately described.	1
Incremental Costs & Outcomes	Compared with usual care, telemonitoring had an estimated ICER of £11,873/QALY, whereas structured telephone support with human-to-human contact had an ICER of £228,035/QALY against telemonitoring. structured telephone support with human-to-machine interface was dominated by usual care.	1
Characterizing Uncertainty	The cost-effectiveness acceptability curve was presented. The chance of telemonitoring being cost-	1

	effective at WTP of £20,000/QALY was 40%. The scenario analysis performed using higher costs of telemonitoring (£215/month) indicated telemonitoring was dominated by structured telephone support with human-to-human contact. Threshold analysis suggested that the monthly cost of telemonitoring has to be higher than £390 to have an ICER greater than £20,000/QALY against structured telephone support with human-to-human contact. Scenario analyses indicated the robustness of the base-case results.	
Characterizing Heterogeneity	Heterogeneity of patients was not addressed	0
**Discussion**		
Study Findings, Limitations, Generalizability & Current Knowledge	Findings, limitations, generalizability, and current knowledge were discussed.	1
**Other**		
Source of Funding	National Institute for Health Research Health Technology Assessment Programme	1
Conflicts of Interest	The salary of an author was supported by the National Institute for Health Research Biomedical Research Unit at the Royal Brompton Hospital.	1

Sandhu *et al.* [10]

Total Quality Score: 21/24

Item	Reported Data	Quality Score (0-1)
**Title & Abstract**		
Title	Cost-effectiveness of implantable pulmonary artery pressure monitoring in chronic heart failure	1
Abstract	A structured abstract with objective, background, methods, results, and conclusion was used. However, the perspective and setting were not specified.	0
**Introduction**		
Background and Objectives	Context and study questions were clear.	1
**Methods**		
Target Population & subgroups	Patients (average age of 62) with NYHA Class III heart failure, hospitalized within 1 year with preserved (21.7%) or reduced ejection fraction (78.3%) were considered.	1
Setting & Location	The setting and location were not clearly specified.	0
Study Perspective	A societal perspective	1
Comparators	CardioMems device versus usual care	1
Time Horizon	Lifetime	1
Discount Rate	Annual discount rate of 3%	1
Choice of Health Outcomes	QALYs	1
Measurement of Effectiveness	The effectiveness inputs were retrieved from the CardioMEMS Heart Sensor Allows Monitoring of Pressure to Improve Outcomes in NYHA Class III Heart Failure Patients (CHAMPION), without detailed description of the clinical trials.	0
Measurement & Valuation of Preference-based Outcomes	The utility values were estimated by converting the 6-month Minnesota Living with Heart Failure questionnaire score for the control arm in the CHAMPION trial into EuroQol- 5 Dimension (EQ-5D) scores	1
Estimating Resources & Costs	The healthcare-related costs were included and estimated from 2014 Medicare Professional Fees	1

	with identified code and the literatures with individual referencing.	
Currency, Price Date & Conversion	2014 USD, using the medical component of the consumer price index	1
Choice of Model	Markov model	1
Assumptions	(1) It was assumed that preventing a hospitalization prevented an inpatient and a two-month post-hospitalization increase in mortality. (2) It was assumed the benefit of the CardioMems device would continue lifelong.	1
Analytic Methods	One-way sensitivity analysis, probabilistic sensitivity analysis, and the subgroup analysis were conducted.	1
**Results**		
Study Parameters	The study parameters and ranges were specified.	1
Incremental Costs & Outcomes	The ICERs for the CardioMems were $71,462 per QALY gained and $48,054 per life-year gained.	1
Characterizing Uncertainty	The cost-effectiveness was most sensitive to the device durability. The study found that 7.3% of simulations showed CardioMems was the preferred intervention at a willingness-to-pay threshold of $50,000, 76.9% at a threshold of $100,000 and 95.1% at a threshold of $150,000	1
Characterizing Heterogeneity	The cost per QALY gained was $82,301 in patients with reduced ejection fraction and $47,768 in those with preserved ejection fraction. In the lower-risk Candesartan in Heart failure: Reduction in Mortality and morbidity (CHARM) cohort, the device would need to reduce heart failure hospitalizations by 41% in order to cost less than $100,000 per QALY gained	1
**Discussion**		
Study Findings, Limitations, Generalizability & Current Knowledge	Findings, limitations, generalizability, and current knowledge were discussed.	1
**Other**		
Source of Funding	Department of Veterans Affair's Quality Enhancement and Research Initiative 04-326	1
Conflicts of Interest	The authors reported the disclosure.	1

Schmier *et al.* [11]

Total Quality Score: 17/24

Item	Reported Data	Quality Score (0-1)
**Title & Abstract**		
Title	Cost-effectiveness of remote cardiac monitoring with the CardioMEMS heart failure system	1
Abstract	No structured abstract was used.	0
**Introduction**		
Background and Objectives	Context and study questions were clear.	1
**Methods**		
Target Population & subgroups	Heart failure patients, without specific age and severity, were considered.	0
Setting & Location	The setting and location were not specified.	0
Study Perspective	The perspective was not clearly specified.	0
Comparators	CardioMEMS versus usual care	1
Time Horizon	5 years	1
Discount Rate	Annual discount rate of 3%	1
Choice of Health Outcomes	QALYs	1

Measurement of Effectiveness	The effectiveness data was estimated from the CHAMPAION trial. The specific description of trial and approach was not presented in the method.	0
Measurement & Valuation of Preference-based Outcomes	The outcomes were retrieved from literatures using three level version of EQ-5D.	1
Estimating Resources & Costs	The estimated costs included implant cost, implant procedure cost, complications cost, routine monitoring, CardioMEMS-related monitoring, HF and non-HF hospitalizations. The values were retrieved from market price, Medicare, and literatures, with detailed description.	1
Currency, Price Date & Conversion	2016 US dollars	1
Choice of Model	Markov model	1
Assumptions	Not all the assumptions were clearly specified in the method.	0
Analytic Methods	One-way sensitivity analysis was conducted.	1
**Results**		
Study Parameters	The study parameters were specified.	1
Incremental Costs & Outcomes	The ICER for the CardioMEMS was $44,832 per QALY.	1
Characterizing Uncertainty	The model was sensitive to the device cost and to whether mortality benefits were sustained and no ICER for any scenarios exceeded $100,000.	1
Characterizing Heterogeneity	The heterogeneity of patients was not addressed.	0
**Discussion**		
Study Findings, Limitations, Generalizability & Current Knowledge	Findings, limitations, generalizability, and current knowledge were discussed.	1
**Other**		
Source of Funding	St. Jude Medical	1
Conflicts of Interest	The authors reported the disclosure.	1

Martinson *et al.* [12]
Total Quality Score: 20/24

Item	Reported Data	Quality Score (0-1)
**Title & Abstract**		
Title	Pulmonary artery pressure-guided heart failure management: US cost-effectiveness analyses using the results of the CHAMPION clinical trial	1
Abstract	A structured abstract with background, objective, methods, results, and conclusion was used, providing complete summary of the study.	1
**Introduction**		
Background and Objectives	Context and study questions were clear.	1
**Methods**		
Target Population & subgroups	Heart failure patients, without specific age and severity, were considered.	0
Setting & Location	US healthcare system	1
Study Perspective	The healthcare payer	1
Comparators	CardioMEMS™ versus usual care	1
Time Horizon	5 years	1
Discount Rate	Annual discount rate of 3%	1
Choice of Health Outcomes	QALYs	1
Measurement of Effectiveness	The effectiveness data was retrieved from CHAMPION trial, with detailed description.	1
Measurement & Valuation of Preference-based Outcomes	US population-based three level version of EQ-5D preference weights	1

Estimating Resources & Costs	The estimated cost was retrieved from Medicare and the Truven Health MarketScan®, with clear description.	1
Currency, Price Date & Conversion	2014 US dollars, based on the consumer price index	1
Choice of Model	Markov model	1
Assumptions	(1) Patients less than 65 years old at implant were assumed to be paid through private insurance, and those 65 years or older at implant were assumed to be paid by Medicare. (2) All implants occurred on a unique scheduled day for each patient, and did not occur during a pre-existing HF hospitalization. (3) the CardioMEMS™ System was assumed to incur a monthly cost of US$ 45 associated with the professional and technical components of reimbursement for remote physiological monitoring. (4) 25% of the standard of care patients also incurred the monthly cost of remote physiological monitoring. (4) The outpatient costs increased due to an improved survival benefit (not due to other changes).	1
Analytic Methods	One-way sensitivity analysis, probabilistic sensitivity analysis, and scenario analysis were conducted.	1
**Results**		
Study Parameters	The parameters were not specified.	0
Incremental Costs & Outcomes	The ICER for treatment group was US$12,262 per QALY.	1
Characterizing Uncertainty	The ICER was sensitive to the time horizon and implant cost. At the WTP of US$ 25,000, >85% of the simulations were cost-effective; >99% were cost-effective at the US$50,000 threshold for the model using HF hospitalization costs only. For the analysis using comprehensive patient management costs, 87% of the simulations were cost-effective at the WTP of US$ 50,000 and >99% were cost-effective at the WTP of US$ 100,000.	1
Characterizing Heterogeneity	The heterogeneity of patients was not addressed.	0
**Discussion**		
Study Findings, Limitations, Generalizability & Current Knowledge	Findings, limitations, generalizability, and current knowledge were discussed.	1
**Other**		
Source of Funding	The source of funding was not specified.	0
Conflicts of Interest	The authors reported the disclosure.	1

Cowie *et al.* [13]

Total Quality Score: 19/24

Item	Reported Data	Quality Score (0-1)
**Title & Abstract**		
Title	The cost-effectiveness of real-time pulmonary artery pressure monitoring in heart failure patients: a European perspective	1
Abstract	A structured abstract with background, objective, methods, results, and conclusion was used. However, the perspective was not specified.	0
**Introduction**		
Background and Objectives	Context and study questions were clear.	1
**Methods**		
Target Population & subgroups	Heart failure patients aged 70, without specific severity, were considered.	0

Setting & Location	European healthcare system	1
Study Perspective	A healthcare payer	1
Comparators	CardioMEMS™ versus usual care	1
Time Horizon	10 years	1
Discount Rate	Annual discount rate of 3%	1
Choice of Health Outcomes	QALYs	1
Measurement of Effectiveness	The effectiveness data was retrieved from CHAMPION trial, without clear description of the trial.	0
Measurement & Valuation of Preference-based Outcomes	EQ-5D data from the patients with CHAMPION trial	1
Estimating Resources & Costs	The estimated costs were the device cost, cost of an implant complication, cost of standard heart failure care, and the cost of heart failure hospitalization, retrieved from Nation Health Service reference costs and literatures.	1
Currency, Price Date & Conversion	The currency, price data & conversion were not specified.	1
Choice of Model	Markov model	1
Assumptions	(1) The patients were assumed to revert back to stable HF after hospitalization. (2) The mortality before 5 years was assumed to be same. (3) 2.7% of the patients were assumed to experience an implant-related complication before entering the model.	1
Analytic Methods	One-way sensitivity analysis, probabilistic sensitivity analysis, and scenario analysis were conducted.	1
**Results**		
Study Parameters	The study parameters were specified.	1
Incremental Costs & Outcomes	The ICER was £19 274 (€24 772) per QALY gained.	1
Characterizing Uncertainty	The results of one-way sensitivity analysis did not dramatically change the results. In the scenario analysis including staff costs, the ICER was £22,342/ QALY. At the WTP of £20,000/ QALY, the probability of the device being cost-effective was 97.6%	1
Characterizing Heterogeneity	The heterogeneity of patients was not addressed.	0
**Discussion**		
Study Findings, Limitations, Generalizability & Current Knowledge	Findings, limitations, generalizability, and current knowledge were discussed.	1
**Other**		
Source of Funding	The source of funding was not specified.	0
Conflicts of Interest	The authors reported the disclosure.	1

Healy *et al.* [14]

Total Quality Score:17 /24

Item	Reported Data	Quality Score (0-1)
**Title & Abstract**		
Title	Wearable cardioverter-defibrillator for prevention of sudden cardiac death after infected implantable cardioverter-defibrillator removal: A cost effectiveness evaluation	1
Abstract	A structured abstract with background, objective, methods, results, and conclusion was used. However, the perspective, setting, and the input in the model were not specified.	0
**Introduction**		

Background and Objectives	Context and study questions were clear.	1
**Methods**		
Target Population & subgroups	Patients, who have undergone implantable cardioverter-defibrillator (ICD) extraction because of device infection and were deemed to require reimplantation due to sudden cardiac arrest (SCA), were considered. But the age of the patients was not shown.	0
Setting & Location	The setting and location were not specified.	0
Study Perspective	The societal perspective	1
Comparators	The wearable cardioverter-defibrillator (WCD) strategy, discharge home, in-hospital stay, and skilled nursing facilities	1
Time Horizon	5 years	1
Discount Rate	Annual discount rate of 3%	1
Choice of Health Outcomes	QALYs and life years	1
Measurement of Effectiveness	The effectiveness data was retrieved from literatures, with clear description and individual referencing.	1
Measurement & Valuation of Preference-based Outcomes	The measurement and valuation of preference-based outcomes were not specified.	0
Estimating Resources & Costs	Direct and indirect cost were both included. Each strategy was clearly specified with the estimated costs to be assigned.	1
Currency, Price Date & Conversion	2014 US dollars, using an inflation rate of 3% to reflect inflation in the consumer price index in accordance with accepted guidelines.	1
Choice of Model	Markov model	1
Assumptions	(1) It was assumed all patients received an ICD after the infection was cleared in the base-case analysis; (2) non-SCA mortality was assumed to be equal in all strategies; (3) patients who did not need ICD were assumed to have a reduced total mortality; (4) it was assumed that use of either a WCD or an ICD further impacted a patient's quality of life.	1
Analytic Methods	Both one-way and two-way sensitivity analyses were conducted.	1
**Results**		
Study Parameters	The study parameters were not clearly specified.	0
Incremental Costs & Outcomes	The ICER of the WCD strategy was $20,300 per life-year or $26,436 per QALY compared to discharge home without a WCD. Discharge to a skilled nursing facility and in-hospital monitoring resulted in higher costs and worse clinical outcomes.	1
Characterizing Uncertainty	The incremental cost-effectiveness ratio was as low as $15,392/QALY if the WCD successfully terminated 95% of SCA events and exceeded the $50,000/QALY WTP threshold if the efficacy was 69%. The WCD strategy remained cost-effective, assuming 5.6% 2-month SCA risk, as long as the time to reimplantation was at least 2 weeks.	1
Characterizing Heterogeneity	The heterogeneity of patients was not addressed.	0
**Discussion**		
Study Findings, Limitations, Generalizability & Current Knowledge	Findings, limitations, generalizability, and current knowledge were discussed.	1
**Other**		
Source of Funding	The source of funding was not specified.	0
Conflicts of Interest	The authors reported the disclosure.	1

References:

1. Burn E, Nghiem S, Jan S, Redfern J, Rodgers A, Thiagalingam A, Graves N, Chow CK. Cost-effectiveness of a text message programme for the prevention of recurrent cardiovascular events. Heart (British Cardiac Society). 2017;103(12):893-4. PubMed PMID: 28235776.
2. Grustam AS, Severens JL, De Massari D, Buyukkaramikli N, Koymans R, Vrijhoef HJM. Cost-Effectiveness Analysis in Telehealth: A Comparison between Home Telemonitoring, Nurse Telephone Support, and Usual Care in Chronic Heart Failure Management. Value in health : the journal of the International Society for Pharmacoeconomics and Outcomes Research. 2018;21(7):772-82. PubMed PMID: 30005749.
3. Cano Martin JA, Martinez-Perez B, de la Torre-Diez I, Lopez-Coronado M. Economic impact assessment from the use of a mobile app for the self-management of heart diseases by patients with heart failure in a Spanish region. Journal of medical systems. 2014;38(9):96. PubMed PMID: 24994514.
4. Mistry H, Gardiner HM. The cost-effectiveness of prenatal detection for congenital heart disease using telemedicine screening. Journal of telemedicine and telecare. 2013;19(4):190-6. PubMed PMID: 23576807.
5. Whetten J, van der Goes DN, Tran H, Moffett M, Semper C, Yonas H. Cost-effectiveness of Access to Critical Cerebral Emergency Support Services (ACCESS): a neuro-emergent telemedicine consultation program. Journal of medical economics. 2018;21(4):398-405. PubMed PMID: 29316820.
6. Nelson RE, Saltzman GM, Skalabrin EJ, Demaerschalk BM, Majersik JJ. The cost-effectiveness of telestroke in the treatment of acute ischemic stroke. Neurology. 2011;77(17):1590-8. PubMed PMID: 21917781.
7. Demaerschalk BM, Switzer JA, Xie J, Fan L, Villa KF, Wu EQ. Cost utility of hub-and-spoke telestroke networks from societal perspective. The American journal of managed care. 2013;19(12):976-85. PubMed PMID: 24512034.
8. Nelson RE, Okon N, Lesko AC, Majersik JJ, Bhatt A, Baraban E. The cost-effectiveness of telestroke in the Pacific Northwest region of the USA. Journal of telemedicine and telecare. 2016;22(7):413-21. PubMed PMID: 26541170.
9. Thokala P, Baalbaki H, Brennan A, Pandor A, Stevens JW, Gomersall T, Wang J, Bakhai K, Al-Mohammad A, Cleland J, Cowie MR, Wong R. Telemonitoring after discharge from hospital with heart failure: cost-effectiveness modeling of alternative service designs. BMJ open. 2013;3(9):e003250. PubMed PMID: 24048626.
10. Sandhu AT, Goldhaber-Fiebert JD, Owens DK, Turakhia MP, Kaiser DW, Heidenreich PA. Cost-Effectiveness of Implantable Pulmonary Artery Pressure Monitoring in Chronic Heart Failure. JACC Heart failure. 2016;4(5):368-75. PubMed PMID: 26874380.
11. Schmier JK, Ong KL, Fonarow GC. Cost-Effectiveness of Remote Cardiac Monitoring With the CardioMEMS Heart Failure System. Clinical cardiology. 2017;40(7):430-6. PubMed PMID: 28272808.
12. Martinson M, Bharmi R, Dalal N, Abraham WT, Adamson PB. Pulmonary artery pressure-guided heart failure management: US cost-effectiveness analyses using the results of the CHAMPION clinical trial. European journal of heart failure. 2017;19(5):652-60. PubMed PMID: 27647784.
13. Cowie MR, Simon M, Klein L, Thokala P. The cost-effectiveness of real-time pulmonary artery pressure monitoring in heart failure patients: a European perspective. European journal of heart failure. 2017;19(5):661-9. PubMed PMID: 28176424.

14. Healy CA, Carrillo RG. Wearable cardioverter-defibrillator for prevention of sudden cardiac death after infected implantable cardioverter-defibrillator removal: A cost-effectiveness evaluation. Heart rhythm. 2015;12(7):1565-73. PubMed PMID: 25839113.
